# Complications Rate and a Multidimensional Analysis of Their Causes of Tube Thoracostomy: A Mixed-Methods Study

**DOI:** 10.7759/cureus.58563

**Published:** 2024-04-18

**Authors:** Kenichiro Takeda, Hajime Kasai, Ikuo Shimizu, Ryutaro Hirama, Nami Hayama, Kohei Shikano, Mitsuhiro Abe, Akira Naito, Takuji Suzuki

**Affiliations:** 1 Department of Respirology, Chiba University Graduate School of Medicine, Chiba, JPN; 2 Department of Medical Education, Chiba University Graduate School of Medicine, Chiba, JPN; 3 Department of Quality and Patient Safety, Chiba University Hospital, Chiba, JPN

**Keywords:** tube thoracostomy, pneumothorax, pleural effusion, mixed-method study, complications

## Abstract

Introduction: Tube thoracostomy (TT) complications are common in respiratory medicine. However, the prevalence of complications and risk factors is unknown, and data on countermeasures are lacking.

Methods: This was a mixed-methods retrospective observational and qualitative study. This retrospective observational study included TT performed on patients admitted to the Department of Respiratory Medicine at our University Hospital between January 1, 2019, and August 31, 2022 (n=169). The primary endpoint was the incidence of TT-related complications. We reviewed the association between complications and patient- and medical-related factors as secondary endpoints. In this qualitative study, we theorized the background of physicians’ susceptibility to TT-related complications based on the grounded theory approach.

Results: Complications were observed in 20 (11.8%) of the 169 procedures; however, they were unrelated to 30-day mortality. Poor activities of daily living (odds ratio 4.3, p=0.007) and regular administration of oral steroids (odds ratio 3.1, p=0.025) were identified as patient-related risk factors. Physicians undergoing training caused the most complications, and the absence of a senior physician at the procedure site (odds ratio 3.5, p=0.031) was identified as a medical risk factor. Based on this qualitative study, we developed a new model for TT complication rates consistent with the relationship between physicians’ professional skills, professional identity, and work environments.

Conclusions: Complications associated with TT are common. Therefore, it is necessary to implement measures similar to those identified in this study. Particularly, a supportive environment should be established for the training of physicians.

## Introduction

Tube thoracostomy (TT) is a medical procedure performed for moderate to severe pneumothorax and pleural effusions [1]. Lung injury, re-expansion pulmonary edema, and infection are commonly encountered severe complications of TT in respiratory medicine [2]. However, the complication rates in previous reports ranged from 11 to 37% [3-10], and the exact complication rates are unknown. Differences in the definition of complications [11], patient characteristics, operators (respiratory physicians, respiratory surgeons, emergency medicine physicians, radiologists), and the medical environment by era and region accounted for the discrepancies [2]. There is a bias towards reports regarding traumatic pneumothorax and a lack of data on other diseases.

Few recent studies have reported approaches to reduce the complications of this procedure from the perspective of medical safety and education [12-14]. However, it should be noted that strategies for patient safety have been attempted without fully investigating the context in which complications of TT occur [2,10]. A detailed survey of the present situation in TT could help develop methods to reduce complications. Thus, we quantitatively analyzed the incidence of complications associated with thoracic drainage and associated risk factors. Furthermore, we conducted a qualitative study to examine the qualifications of medical personnel involved in cases of complications and to explain the background through theories or models.

## Materials and methods

Population and design

**Figure 1:**
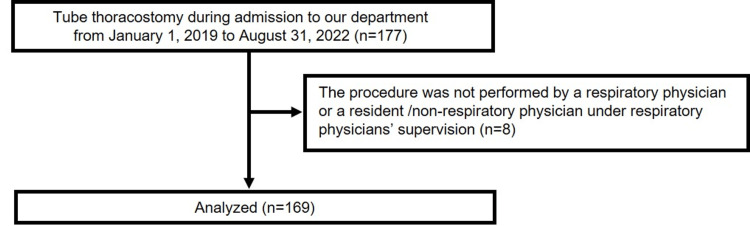
Flow chart for participant selection. All 177 tube thoracostomy procedures were performed on patients admitted between January 2019 and August 2022. Emergency doctors performed eight procedures, and these were excluded from the analysis in accordance with the exclusion criteria.

We conducted a mixed-methods retrospective observational and qualitative study. This retrospective observational study included TT performed on patients with pneumothorax or pleural effusion admitted in the Department of Respiratory Medicine at our University Hospital between January 1, 2019, and August 31, 2022. Our University Hospital is a national hospital with 850 beds. We selected 177 consecutive procedures from the medical records, and some were excluded from the study based on the following criteria: (1) the patient was <16 years old; (2) the procedure was not performed by a respiratory physician or a resident/non-respiratory physician under the supervision of a respiratory physician; (3) the patient refused to participate in the study through public documentation released on the website; and (4) patients’ geographics or procedure details were unavailable from the medical records. Finally, 169 procedures (126 cases) were analyzed (Figure 1). To collect quantitative data, the sample size for a single-center study was determined using a study with similar backgrounds [3]. This study estimated a complication rate of 11%, and a minimum sample size of 151 procedures was required to achieve a 95% confidence interval of 6-16%.

This retrospective study showed that most TT-related complications occurred during procedures performed by residents and fellows. Additionally, the results suggested that performing procedures without a senior physician or outside working hours is a complication risk factor. There are multiple processes to reach the skill level from novice to expert [15]; however, an educational method for physicians in TT training has not yet been established. Owing to many uncertainties, an exploratory approach was deemed necessary, and a qualitative study was conducted to assess the qualifications of physicians in training in this situation. In Japan, before beginning each specialty training, all residents rotate internal medicine, including respiratory medicine, for ≥6 months in a two-year residency program [16]. Additionally, respiratory physicians can obtain a specialty in the seventh year after graduation at the fastest rate by completing respiratory specialty training [17]. In this study, semi-structured focus group interviews (FGIs) were conducted with respiratory physicians in the eighth year of post-graduation at our University Hospital. FGIs were conducted by a respiratory physician (KT) and supervised by a medical safety doctor (IS). For FGIs, we explained that it was not a place to blame for complications, and we made efforts to proceed in a manner that ensured psychological safety.

This study was conducted by the Declaration of Helsinki and was approved by the Ethics Committee of Chiba University Hospital (Approval No. HK202301-08). All participants in this retrospective study were free to opt out of participating in the study. Additionally, all participants in the qualitative study provided written informed consent at the beginning of the FGIs. This study adhered to the STROBE and SRQR guidelines.

Data collection

Quantitative data were collected retrospectively from the medical records. The incidence of TT-related adverse events was the primary endpoint. This study defined complications as acute and relatively severe events associated with drain insertion procedures. Additionally, we referred to previous reports and categorized complications as follows: chest injury, extra-thoracic insertion, kinked or obstructed drain (within 24 h), infection at the insertion site (within seven days), spontaneous dislodgement, symptomatic subcutaneous emphysema, and others. Furthermore, we reviewed the association between adverse events and patient- and medical-related factors as secondary endpoints. The extracted patient-related factors included age, sex, activities of daily living (ADL), comorbidities, concomitant medications, oxygen therapy, body size, vital signs, and serum albumin. Medical factors included operator experience, preoperative examination, devices used, and operating environment. Furthermore, 30-day survival was examined to confirm whether complications affected short-term outcomes.

Qualitative data were collected from semi-structured FGIs conducted by respiratory physicians. The respiratory physicians were asked the following questions: (Q1) What do you think is necessary to prevent complications during TT? (Q2) Why did you not call your supervisors outside working hours? (Q3) Why was TT not delayed until the following morning? (Q4) What were your circumstances when you performed TT outside of working hours? Two researchers skilled in qualitative research (HK and IS) validated the interview guide before data collection.

Data analysis

Quantitative data are expressed as mean ± standard deviation (SD) unless otherwise indicated. The Mann-Whitney U test was used to compare the group in which complications occurred with the other group. Statistical significance was set at P < 0.05. All statistical analyses were performed using JMP 15.0 (Cary, North Carolina, USA).

For qualitative analysis, grounded theory methodology was selected to theorize the background of the development of complications. Based on grounded theory methods, we repeated the sampling and coding until we saturated the concept and developed a theory [18]. KT and HK independently read and coded all transcripts. Codes with similar concepts were reviewed and categorized. During coding, a continuous comparative analysis was performed within and between categories. Following the constant modification of the categories, a model suitable for the research question was generated. IS supervised this qualitative analysis.

## Results

Quantitative study

Complications were observed in 20 (11.8%) of 169 procedures. Table 1 shows the details of the complications. A kinked or obstructed drain (within 24 h) was the most frequent complication (n=6 (3.6%)), and all patients required additional TT for untreated pneumothorax. However, complication-related deaths were not reported, and no significant correlation was found between adverse events and 30-day mortality (Table 2). Although there were no differences in the complication rates between pneumothorax and pleural effusion or underlying diseases (malignancy, emphysema, and interstitial pneumonia), complications were significantly more frequent in patients with poor ADL (inability to walk independently) (odds ratio 4.3, p=0.007) and regular administration of oral steroids (odds ratio 3.1, p=0.025). Kinked or obstructed drains were observed with a significantly higher frequency in patients receiving steroids (n=4, p=0.029).

**Table 1:** Details of the complications in tube thoracostomy insertion and operators’ rank.

	Total	Resident and fellow	Senior physicians
Total procedures, n	169	156	13
Complications, n (%)	20 (11.8)	19 (12.2)	1 (7.7)
Chest injury	1 (0.6)	1 (0.6)	0 (0)
Extra-thoracic insertion	3 (1.8)	2 (1.3)	1 (7.7)
Kinked or obstructed drain (within 24 h)	6 (3.6)	6 (3.8)	0 (0)
Infection at the insertion site (within seven days)	0 (0)	0 (0)	0 (0)
Spontaneous dislodgement	4 (2.4)	4 (2.6)	0 (0)
Symptomatic subcutaneous emphysema	4 (2.4)	4 (2.6)	0 (0)
Adverse events due to premedication	2 (1.2)	2 (1.3)	0 (0)

**Table 2:** Association of complications in tube thoracostomy insertion with patient and medical factors. *Poor ADL was defined as the inability to walk independently. ADL: actives of daily living.

	Total	Complication	Non-complication	P-value
Total procedures, n (%)	169	20 (11.8)	149 (88.2)	NA
Age, median (range)	71 (16-87)	68 (43-86)	71 (16-87)	0.575
Male sex, n (%)	121 (71.6)	13 (65.0)	108 (72.5)	0.598
Diagnosis, n (%)				0.812
Pneumothorax	105 (62.1)	12 (60.0)	93 (62.4)	
Idiopathic	4 (2.3)	0 (0)	4 (2.7)	
Secondary	101 (59.8)	12 (60.0)	89 (59.7)	
Traumatic or iatrogenic	0 (0)	0 (0)	0 (0)	
Pleural effusion	64 (37.9)	8 (40.0)	56 (37.6)	
Carcinomatous pleurisy	39 (23.1)	4 (20.0)	35 (23.5)	
Infectious pleurisy/empyema	9 (5.3)	1 (5.0)	8 (5.4)	
Empyema with fistula	11 (6.5)	3 (15.0)	8 (5.4)	
Others	5 (3.0)	0 (0)	5 (3.4)	
Comorbidities				
Malignancy	69 (40.8)	8 (40.0)	61 (40.9)	1.000
Emphysema/lung cyst	46 (27.2)	7 (35.0)	39 (26.2)	0.428
Interstitial lung disease	70 (41.4)	10 (50.0)	60 (40.3)	0.472
Poor ADL^*^, n (%)	28 (16.6)	8 (40.0)	20 (13.4)	0.007
Medication, n (%)				
Oral steroids	40 (23.7)	9 (45.0)	31 (20.8)	0.025
Antiplatelet	20 (11.8)	3 (15.0)	17 (11.4)	0.710
Anticoagulant	13 (7.7)	3 (15.0)	10 (6.7)	0.187
Non-use of fluoroscopy, n (%)	14 (8.3)	3 (15.0)	11 (7.4)	0.219

Table 1 shows that almost all complications occurred during the procedures performed by the residents (one to two years of medical experience) or fellows (three to six years of medical experience). Table 3 shows the results of the subgroup analysis. Complications increased significantly, especially for procedures performed without a senior physician (with at least seven years of medical experience) (odds ratio 3.5, p=0.031). Additionally, there was a trend towards increased complications when the procedures were performed outside working hours (odds ratio 2.7, p=0.094), without an assistant (odds ratio 4.1, p=0.080), and the disuse of fluoroscopy (odds ratio 3.0, p=0.134). The lack of preprocedural examinations (x-ray, computed tomography, and ultrasonography) was not associated with complications.

**Table 3:** Associations between complications and medical side factors when the operator was a resident or fellow

	Total	Complication	Non-complication	P-value
Total procedures, n (%)	156	19 (12.2)	137 (87.8)	NA
Absence of supervision by a senior physician, n (%)	22 (14.1)	6 (31.6)	16 (11.7)	0.031
Procedures at outside of working hours, n (%)	26 (16.7)	6 (31.6)	20 (14.6)	0.094
Absence of an assistant, n (%)	9 (5.8)	3 (15.8)	6 (4.4)	0.080
Non-use of fluoroscopy, n (%)	11 (7.1)	3 (15.8)	8 (5.8)	0.134

Qualitative study

Six respiratory physicians (two interviews with three physicians each) participated in the interviews based on previous results. Each interviewed respiratory physician had worked in a different hospital and had been affiliated with our hospital for a year.

**Figure 2:**
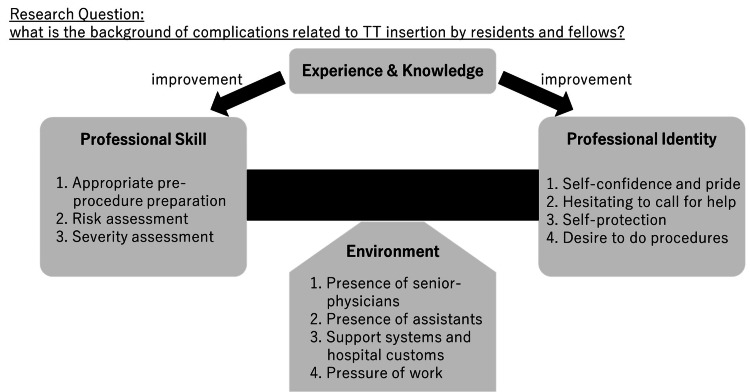
A model of the background of complications related to tube thoracostomy by physicians during training We attempted to resolve the following research question: What is the background of complications related to tube thoracostomy (TT) by residents and fellows by analyzing the interview results? Skills are important for completing TT without complications. Additionally, professional identity plays a significant role in the decision to perform a procedure. Thus, we hypothesized that an imbalance between skill and identity may cause these complications. Most interviewees agreed that their professional skills and identity could be strengthened by increasing their experience and knowledge. Furthermore, the interviews and quantitative data results indicate that environmental factors protected residents and others from developing complications. Thus, we hypothesized that the imbalance between skills and identity is appropriately compensated in a well-organized environment.

We developed a model in which complications were more likely to occur due to an imbalance between physicians’ professional skills and professional identity (Figure 2). Experience and knowledge enhance professional skills and knowledge. Environmental factors support this balance. Based on FGIs, we explain why we support this model. The questions and typical answers in the FGIs are shown in Table 4.

**Table 4:** The questions and typical answers in focus group interviews. TT: tube thoracostomy.

Questions	Typical answers
Q1. What do you think is necessary to prevent complications during TT?	“Increasing experience and knowledge improved skills and self-confidence.”
Q2. What were the reasons you did not call your supervisors outside of working hours?	“My supervisor might get angry if I call him.” “I wish my supervisor would rest during off hours.” “My pride for the profession got so high that I could not call for help”
Q3. Why the TT was not delayed until the following morning?	“I dislike problems caused by not taking procedures more than complications.”
Q4. What were the surrounding circumstances when you performed TT outside working hours?	“It was decided that an on-call physician should not call another respiratory physician for support.”

First, all participants indicated that increasing their experience and knowledge improved their skills and self-confidence in Q1. Furthermore, they determined the urgency and safety of the procedure and conducted thorough preparations before it as a necessary skill.

Second, they were found to have psychological barriers to consulting their supervisors for help in Q2 and Q3. We hypothesized that this is closely related to professional identity. One aspect of this factor was reticence toward supervisors. Many participants also stated that they would perform the TT even if they thought the patient's condition was stable and could be observed until the next morning. Particularly, patients with pneumothorax may present with emergency conditions such as respiratory failure or tension pneumothorax. Therefore, observation was not an acceptable option for the fellows, and performing TT helped them gain a sense of security. It has been suggested that a psychological desire to protect oneself from trouble underlies this. While positive opinions about self-confidence and pride were common, these could also be psychological barriers to calling for help from supervisors. 

Finally, we examined physicians’ environments to explain the increase in complications in specific situations, such as outside working hours. Fellows were stressed about the absence of supervisors or assistants outside their working hours. In addition, they were busy at work, and the support system for physicians during training might have been insufficient. The FGI clearly showed that complications increased when physicians in training were placed in unfavorable environments. Paradoxically, this means that the complications experienced by residents and fellows decreased when they accessed various types of support. Thus, the environment is considered protective for inexperienced professionals.

## Discussion

This study is valuable for identifying new patient-side complication risk factors of TT. In this study, the frequency of complications was significantly increased in patients with poor ADL who regularly receive oral steroids. Poor ADL may have caused postural disadvantages during TT implementation and contributed to increased complications. Furthermore, excessive tension in the chest tube during daily care in inpatient wards may induce spontaneous dislodgement. Patients administering oral steroids have significantly increased kinked or obstructed drains; however, the cause remains unknown. No local infections were observed in this study. Although a previous study also reported that obesity increases the complications of traumatic pneumothorax [10], the missing data were too large to be analyzed in our study. However, studies on patient-side factors and TT complications are limited. Recognizing cases with a high risk of complications can increase the safety awareness of medical personnel and reduce complications.

To the best of our knowledge, this is the first report of a mixed-methods study of TT complications. However, several reports have discussed the medical factors related to TT complications. Our findings can be compared to the framework of contributory factors affecting clinical work reported by Vincent et al [19]. Our definition of professional skill and identity corresponds to individual factors (knowledge and skill), and our definition of the environment corresponds to individual factors (mental and physical health), team factors, work factors, and environmental, organizational, and management factors. Physicians’ lack of knowledge and skills during training is an obvious risk factor; therefore, supporting it with other factors is a reasonable countermeasure.

Based on the results of this study, it would be important to create an environment in which physicians can safely gain experience with procedures to enhance their professionalism. The findings of this study will help formulate countermeasures for medical safety. Santos et al. reported that a quality improvement initiative applied to TT can reduce complications [12]. Their initiatives, direct supervision during TT and mandatory training, are consistent with our findings. They also attempted to scandalize the procedure. The variation in procedures among physicians in TT has been pointed out [20], and some of our interviewees also pointed this out. Although this study did not determine any disadvantages due to variations in the procedures, establishing a standardized procedure may be useful for evaluating operator skills and teaching physicians during training. Scales that measure physicians’ TT skills have also been reported [21,22] and can be used to determine proficiency in the procedure. Furthermore, simulator-based education has been reported as a safe teaching method for patients [13].

This study has some limitations. First, it was a single-center study. Owing to the characteristics of our facility, there are very few cases of idiopathic pneumothorax in young patients, whereas there are many cases of secondary pneumothorax with underlying diseases. Similarly, the frequency of complications and their breakdown may differ depending on the differences between the patient population and that of a general city hospital. However, each respiratory physician who participated in the interviews had experience at different training hospitals. Therefore, we believe that the generalizability of the results obtained from this qualitative study is ensured. Secondly, this was a retrospective study. Unquestionably, the prevalence of complications may have been underestimated due to insufficient extraction of complications from the medical records. References to previous reports also suggest the existence of risk factors that were not fully evaluated in this study. Further prospective studies are required for a more detailed examination.

## Conclusions

The frequency of complications associated with TT was 11.8%. New risk factors for complications have been identified. Furthermore, a survey of the complication rates occurring during TT performed by physicians in training revealed the need to improve the medical environment, especially after working hours. Safety measures based on these findings should be implemented to reduce the frequency of complications further. Particularly, a supportive environment should be created to train physicians.
